# Cost and affordability implications of transitioning from current diets to National dietary guidelines and EAT-Lancet recommendations in Argentina: a modelling study

**DOI:** 10.1186/s12937-025-01212-7

**Published:** 2025-10-22

**Authors:** Florencia Cámara, Leila Guarnieri, María Victoria Tiscornia, Stefanie Vandevijvere, Sally Mackay, Boyd Swinburn, Luciana Castronuovo

**Affiliations:** 1 Fundación Interamericana del Corazón Argentina (FIC Argentina), Autonomous City of Buenos Aires, Argentina; 2 Sciensano, Brussels, Belgium; 3 Faculty of Medical and Health Sciences, Epidemiology and Biostatistics, University of Auckland, Auckland, New Zealand

**Keywords:** Cost, Affordability, Diets, Argentina, Eat-Lancet planetary health

## Abstract

Current dietary patterns contribute to health issues and pose high demands on the food production system, leading to environmental degradation. This paper aims to analyze the cost and affordability of current diets in Argentina, compared to one diet based on National Dietary Guidelines and 3 variants of diets based on EAT-Lancet Recommendations. Methods: The methodology proposed by INFORMAS (International Network for Food and Obesity/Non-communicable Diseases Research, Monitoring and Action Support) was used to design six model diets for a reference household, considering the most consumed food products by the Argentinean population and similar healthier and/or more environmentally sustainable options. Nutritional information and prices of food products were obtained from official sources. Monte Carlo Simulations were performed to estimate the average cost of the diets (and variability). Affordability was measured as the percentage of average monthly household income each model diet represents. Results: On average the diet based on National Dietary Guidelines was the most expensive (274.95 USD; 95% CI: 274.85–275.05), followed by the current diet (261.84 USD; 95% CI: 261.62–262.06), the flexitarian diet that includes higher amount of animal protein sources (design to be more similar to the current consumption pattern in Argentina) (259.43 USD; 95% CI: 259.30–259.55), and then the vegan diet (256.96 USD; 95% CI: 256.90–257.03). The lowest costs were found for the current isocaloric diet (248.29 USD; 95% CI: 248.06–248.52) and the flexitarian diet with less animal proteins (248.37 USD; 95% CI: 248.26–248.48). Between 53% and 59% of the average income is needed to cover the cost of diets. Conclusions: Diets based on National Dietary Guidelines are on average the most expensive (least affordable), while the least expensive (most affordable) are the current isocaloric diet and the flexitarian diet with fewer animal protein sources, suggesting that there are dietary options that can reduce greenhouse gas emissions and provide health benefits without increasing food expenses.

**Supplementary Information:**

The online version contains supplementary material available at 10.1186/s12937-025-01212-7.

## Background

Unhealthy dietary choices and habits are the main determinants of malnutrition, including the concomitance of overweight, micronutrient deficiencies, and non-communicable diseases (NCDs) [1]. Current dietary patterns contribute to health issues and pose high demands on the food production system, leading to environmental degradation [2, 3] so it is important to add environmental sustainability considerations to diet planning [2, 4]. In the 2019 Special Report, the Intergovernmental Panel on Climate Change (IPCC) emphasized that embracing healthy and sustainable diets provides significant opportunities to reduce greenhouse gas emissions from food systems and enhance health outcomes, preserving long-term food security [5]. The current global food system is conservatively estimated to cost nearly USD 20 trillion annually, primarily due to its extensive negative effects on both human health and the environment [6].

Scientists of the EAT-Lancet Commission on Healthy Diets From Sustainable Food Systems developed global targets and recommendations for an EAT-Lancet Planetary Diet, based on all the available nutritional and environmental evidence, to promote healthy diets for sustainable food systems [3]. Following that, the most affordable way to meet this target was estimated in 159 countries and it was found that the average EAT–Lancet diet is affordable in high-income countries. Still, it is not affordable for the poorest world population (in low and medium income countries), and it is more expensive than the food combination that meets the nutrient adequacy at the minimum cost [7]. The transition to healthier and more environmentally sustainable food systems requires strong policy actions, with cultural acceptability, as well as cost and affordability, being two key dimensions for achieving this.

Modeling studies that took the approach of planetary health diets resulted in nutritionally sufficient diets [8, 9] similar to existing dietary patterns, such as the Mediterranean diet, which could be feasible [10]. The academic evidence regarding the costs of healthy and environmentally sustainable diets is still varied; some studies found that it is possible to access a healthy and more sustainable diet with less cost [11–13], yet other authors concluded that households would incur higher costs to follow sustainable healthy diets [14–16]. However, the comparability of results between countries is limited due to methodological differences, the indicators and data sources used to define a sustainable diet, and differences in the local economic and food system context.

The Argentine dietary pattern consists of high consumption of red meat and ultra-processed foods (UPF), and low consumption of legumes, whole grains, fresh fruit, and vegetables [17]. The cost and affordability of diets have been identified as an important barrier to accessing a healthy diet [18]. Previous studies in Argentina found that healthy diets are on average more expensive than current diets [13, 19]. Specifically, a modeling study found that households need to spend, on average, 32% more money on food to guarantee the same energy intake from a healthy diet compared to the current diet [19]. Research on healthy and sustainable diets is emerging in the region [11, 20, 21] and in the country, with recent studies suggesting that healthy and more sustainable diets [22, 23] could be reached at a similar and even lower cost than the current diet [13], however, this may face considerable challenges due to prevailing cultural consumption habits [22].

Due to the current gaps in the study and recommendation of healthy, sustainable, and affordable diets adapted to the Argentine culture and habits, the objective of this study was to analyze the cost and affordability of current diets in Argentina, compared to other diets based on National Dietary Guidelines and EAT-Lancet Recommendations.

## Methods

The research followed the optimal approach methodology proposed by INFORMAS (International Network for Food and Obesity/Non-communicable Diseases Research, Monitoring and Action Support) [24]. INFORMAS is a global network of organizations and researchers committed to the promotion of healthy food environments worldwide, with the ultimate goal of reducing obesity, diet-related NCDs, and their related inequalities. The INFORMAS protocol provides guidelines to systematically collect and analyze information on diet cost and affordability in a reproducible and comparable way.

### Design of different diet models

Six model diets were developed for a reference household (composed of four people: a 45-year-old man and woman, a 7-year-old girl, and a 14-year-old boy): current diet (CD), healthy diet (HD), three variants based on EAT-Lancet recommendations, being two flexitarian scenarios (FD1 and FD2) and one vegan diet (VD), and a current isocaloric diet (CID). The design of CD and CID was based on the current dietary pattern of the Argentinean population [25]; HD was based on the recommendation of the National Dietary Guidelines (GAPA, spanish acronym) [26]; and the three variants of EAT-Lancet diets were based on the dietary recommendations from the EAT-Lancet Commission on Healthy Diets from Sustainable Food Systems [3], with some adaptations for the local context. Given that these guidelines establish some flexibility by specifying ranges in grams for the food groups to be included in the diets, the FD1 was designed to include a higher amount of animal protein sources (meats and eggs) and dairy products, in order to have a version of the flexitarian diet more similar to the Argentinean consumption pattern. Meanwhile, the FD2 includes smaller amounts of these food groups, and the VD excludes them altogether. Conversely, the VD provides a higher amount of legumes, and unsaturated oils-nuts and seeds compared to the FD1 and FD2 (see Table 1).

**Table 1:** Energy, nutrient and food groups targets of diets

Model diets	Current Diet (CD)	Current Isocaloric Diet (CID)	Healthy Diet (HD)	EAT Lancet Diets		
				Flexitarian Diet 1 (FD1)	Flexitarian Diet 2 (FD2)	Vegan Diet (VD)
Energy (kcal/day/person)	Energy requirements for current BMI and physical activity based on ENNyS (± 1.5%): M 2856; W 2048; B 3212; G 1711	Energy requirements for healthy BMI and recommended physical activity (± 1.5%): M 2695; W 2013; B 2891; G 1560				
Carbohydrates (%kcal)	ENNyS mean: M 48; W 52; B 51; G 54		GAPA recommendation: 55			
Protein (%kcal)	ENNyS mean: M 17; W 16; B 16; G 15		GAPA recommendation:15			
Total Fat (%kcal)	ENNyS mean: M 34; W 34; B 34; G 34		GAPA recommendation: 30			
Saturated Fat (% kcal)	ENNyS mean: M 11; W 11; B 11; G 12		GAPA recommendation < 10			
Free Sugars (%kcal)	ENNyS mean: M 17; W 21; B 19; G 23		GAPA recommendation < 10			
Fiber (g/day/person)	ENNyS mean: M 13; W 11; B 13; G 10		GAPA recommendation M > 33; W > 25; B > 36; G > 19			
Sodium (mg/day/person)	ENNyS mean: M 1891; W 1380; B 2145; G 1503		GAPA recommendation M < 2000; W < 1500; B < 2200; G < 1200			
Fruits and vegetables (grams/day/2000 kcal)	ENNyS mean: M 188; W 257; B 130; G 157		GAPA recommendation: 700	EAT Lancet recommendation: Fruits 300; Vegetables 400	EAT Lancet recommendation: Fruits 250; Vegetables 450	EAT Lancet recommendation: Fruits 250; Vegetables 450
Tubers or starchy vegetables (grams/day/2000 kcal)	ENNyS mean: M 212; W 205; B 222; G 213		GAPA recommendation: 370	EAT Lancet recommendation: 80	EAT Lancet recommendation: 80	EAT Lancet recommendation: 80
Grains (grams/day/2000 kcal)				EAT Lancet recommendation: 220	EAT Lancet recommendation: 220	EAT Lancet recommendation with Argentinean adaptation: 350
Legumes (grams/day/2000 kcal)				EAT Lancet recommendation: 50	EAT Lancet recommendation: 70	EAT Lancet recommendation: 100
Dairy Foods (grams/day/2000 kcal)	ENNyS mean: M 108; W 134; B 161; G 255		GAPA recommendation: 530	EAT Lancet recommendation: 460	EAT Lancet recommendation: 295	EAT Lancet recommendation:0
Animal protein sources (grams/day/2000 kcal)*	ENNyS mean: M 226; W 196; B 180; G 140		GAPA recommendation: 155	EAT Lancet recommendation: 100	EAT Lancet recommendation: 60	EAT Lancet recommendation:0
Unsaturated oils - Nuts and seeds (grams/day/2000 kcal)	ENNyS mean: M 22; W 23; B 20; G 19		GAPA recommendation: 30	EAT Lancet recommendation: 50	EAT Lancet recommendation: 70	EAT Lancet recommendation: 90
Discretionary foods (%kcal)	ENNyS mean: M 34; W 36; B 40; G 42		GAPA recommendation: <13.5%	EAT Lancet recommendation: < 8.6	EAT Lancet recommendation: < 8.6	EAT Lancet recommendation: < 8.6
Non-sugary Beverages (ml/day/2000kcal)	ENNyS mean: M 1842; W 2375; B 1229; G 1113		GAPA recommendation: 2000	GAPA recommendation: 2000	GAPA recommendation: 2000	GAPA recommendation: 2000
Alcohol (grams/day/2000 kcal)**	ENNyS mean: M 108; W 37		0	0	0	0
Supplement (Vitamin B12)	N.A.	N.A.	N.A.	N.A.	N.A.	1 dosis (1000mcg/person/day)

*Includes beef, lamb, pork, chicken and other poultry, fish and egg

** Only included in the diet of adults. M, Male; W, Women, B, Boy; G, Girl; BMI, Body Mass Index; ENNyS, Second National Nutrition Survey; GAPA, National Dietary Guidelines of Argentina; N.A., Not applicable

For designing the model CD a list of the most commonly consumed foods was obtained, defined as those food products that were reported to be consumed by at least 2% of the population in the Second National Nutrition and Health Survey, conducted in 2018 (ENNyS2, Spanish acronym) [27], the most recent data source. The selection ensures enough food variability within each food group used in the GAPA.

For the HD, FD1, FD2, and VD, food and beverage items were added as necessary following the normative requirements. For example, within the group ‘legumes, cereals, potato, bread and pasta’ brown rice and whole wheat flour were incorporated into the HD, and plant-based proteins such as lentils and beans were added for the FD1, FD2, and VD models. In the case of the VD model, vitamin B12 supplementation was included as this is a critical nutrient in diets with low or no intake of animal-based foods [3]. These additions made it possible to have a wide range of food products within each food group and to achieve energy and nutritional targets. Qualitative adjustments were conducted by two nutritionists.

The final food list (*n* = 124) was classified into 25 groups as defined in the INFORMAS Protocol [24] (see Table A.1 in Additional file 1).

### Nutritional information

The nutritional information for each of the food products included in the model diets was completed considering the following variables: Energy kcal/100 g; Fat g/100 g; Sat fat g/100 g; Carbohydrates (CHO) g/100 g; Free sugars g/100 g; Fibre g/100 g; Protein g/100 g; Sodium mg/100 g. The data source used was the database developed for the analysis of the ENNyS2 [27].

### Energy, nutrient, and food group targets

The CD daily energy consumed per member of the reference household was calculated based on the mean body weight and current physical activity following the methodology of a similar study [14]. The values corresponding to nutrient and food group targets were estimated based on an analysis of the 24-hour recall reported in the ENNyS2. For the HD, FD1, FD2, and VD, the daily energy consumed per member was calculated based on the recommended BMI and physical activity level. The estimation of the nutrient requirements was based on GAPA. The values corresponding to food group targets were based on GAPA for the HD and based on the EAT-Lancet recommendations for FD1, FD2, and VD.

In order to have a variability range and, thus, yield different combinations of diets, minimum and maximum ranges of energy and nutrients consumed by each member were established as per the INFORMAS protocol [24], which allows for a +/− 30% margin for nutrient requirements and +/− 1.5% for energy yields.

For the CID, only the amount of energy provided by the diet was adjusted. It was modeled considering the same energy intake as for the HD, FD1, FD2, and VD, but the nutrient and food group targets were the same as in the CD (see Table 1).

Three versions of each model diet were designed, considering variations in the biweekly combination of food products that meet the defined energy, nutrients, and food group targets for each member of the reference household. Three versions of each diet were sufficient to be able to apply a Monte Carlo simulation, using a combination of each version per food group.

### Product prices, diet costs, and affordability

Food product prices were obtained from a web scraping from the Precios Claros Web [28], and referred to April/May 2020, the most recent data source available at the time of the estimation. Precios Claros is a website developed by the National Government of Argentina to offer an Electronic Price Advertising System that is continually updated. The website provides information on product prices in different retailers and wholesalers throughout the country, informing the population so they can make price comparisons for their entire shopping list in various nearby stores and thus be able to choose where and how to buy better.

Data curation of the entire dataset was performed in Python 3.9 and included the following steps: (a) identifying the location of retailers or wholesalers and classifying them by region; (b) identifying the food products included in the diets; (c) analysing the price distribution of each food product; (d) cleaning up outliers and errors detected in the dataset; (e) calculating the average price per food product in the Greater Buenos Aires Area. The average price was calculated for each food product included in the diets, considering information of approximately 130 retailers and wholesalers located in the Greater Buenos Aires Area, where almost 40% of the country’s population lives. Then, the cost of the diets was calculated as the sum of the product of the average estimated food price and the amount of the food product included in the diet, by food group.

A Monte Carlo simulation was performed to estimate the average cost of each model diet. The simulation used the cost of the 24 food groups in the three versions designed of each model diet to combine them and to obtain 10000 possible combinations per model diet, also called iterations, which generate a distribution of 10000 possible total costs for each model diet. Then, the mean, standard deviation and confidence interval of each model diet cost distribution were estimated. The estimated average cost is reported in Argentine pesos (AR$) per household biweekly, in US Dollars (USD) per household biweekly, and in USD per person (assimilated to a 45-year-old woman with an intake of 2000 kcal per day) daily. An exchange rate of AR$ 64.44 per USD was used in the currency conversion.

Affordability was measured as the percentage of the monthly average household income that each model diet cost represents. The Permanent Household Survey [29] (second quarter of 2020) was used for estimating average household income.

To analyze the cost and energy intake contribution by food groups in each model diet, the initial 25 food groups were re-grouped into 11 food groups following EAT-Lancet [3], with minor adaptations to respect the local context in Argentina (see Table A.1 in Additional file 1).

## Results

Table 2 shows the percentage contribution of each food group to energy intake in each of the diet models. In the CD and CID models, the groups that contributed the most to total energy intake are discretionary foods, accounting for approximately one-third of the calories in both cases, followed by grains (22.3% and 22.2% respectively) and animal protein sources (15.5% and 15.0% respectively). In the HD, FD1, FD2, and VD models, the groups that contributed the most to the total energy intake are grains (25.3%, 29.2%, 30.1%, and 29.8% respectively), followed by unsaturated oils - nuts and seeds (16.7%, 23.5%, 27.6%, and 31.7% respectively), and fruits and vegetables (15.7%, 16.2%, 17.1%, and 17.3% respectively).

**Table 2:** Energy intake, and food group contribution to energy intake

Model diets	Current Diet (CD)	Current Isocaloric Diet (CID)	Healthy Diet (HD)	EAT Lancet Diets		
				Flexitarian Diet 1 (FD1)	Flexitarian Diet 2 (FD2)	Vegan Diet (VD)
Energy intakes in kcal, per household biweekly	138880	129570	129010	128637	127979	128251
Energy intakes in kcal, per household daily	9920	9255	9215	9188	9141	9161
Food groups - contribution to energy intake, in %						
Fruits and vegetables	3.7	3.8	15.7	16.2	17.	17.3
CI 95%	(3.7–3.7)	(3.8–3.8)	(15.7–15.7)	(16.2–16.2)	(17.1–17.1)	(17.3–17.3)
Tubers or starchy vegetables	2.5	2.4	2.2	3.8	3.8	3.6
CI 95%	(2.5–2.5)	(2.4–2.4)	(2.2–2.2)	(3.8–3.8)	(3.8–3.8)	(3.6–3.6)
Grains	22.3	22.2	25.3	29.2	30.1	29.8
CI 95%	(22.3–22.3)	(22.2–22.2)	(25.3–25.3)	(29.2–29.2)	(30.1–30.1)	(29.8–29.8)
Legumes	0.7	0.8	6.3	4.3	6.5	14.8
CI 95%	(0.7–0.7)	(0.8–0.8)	(6.3–6.3)	(4.3–4.3)	(6.5–6.5)	(14.8–14.8)
Dairy foods	9.9	10.0	13.5	12.5	7.9	0.0
CI 95%	(9.9–9.9)	(10.0–10.0)	(13.5–13.5)	(12.5–12.5)	(7.9–7.9)	N.A.
Animal protein sources	15.5	15.0	12.3	8.1	4.6	0.0
CI 95%	(15.4–15.5)	(15.0–15.1)	(12.3–12.3)	(8.1–8.1)	(4.6–4.6)	N.A.
Unsaturated oils - Nuts and seeds	11.2	10.5	16.7	23.5	27.6	31.7
CI 95%	(11.2–11.2)	(10.4–10.5)	(16.7–16.7)	(23.5–23.5)	(27.6–27.6)	(31.7–31.7)
Discretionary foods	32.9	34.0	8.0	2.4	2.4	2.7
CI 95%	(32.9–33.0)	(33.9–34.0)	(8.0–8.0)	(2.4–2.4)	(2.4–2.4)	(2.7–2.7)
Non-sugary beverages	0.0	0.1	0.1	0.1	0.1	0.1
CI 95%	(0.0–0.0)	(0.1–0.1)	(0.1–0.1)	(0.1–0.1)	(0.1–0.1)	(0.1–0.1)
Alcohol*	1.2	1.3	0.0	0.0	0.0	0.0
CI 95%	(1.2–1.2)	(1.3–1.3)	N.A.	N.A.	N.A.	N.A.
Supplement**	0.0	0.0	0.0	0.0	0.0	0.0
CI 95%	N.A.	N.A.	N.A.	N.A.	N.A.	N.A.

*Alcohol products were only included in the current diet of adults

**Supplements were only included in the vegan diet. *CI* Confidence Interval; *N.A.* Not applicable. Source: Own estimation

When analyzing the cost of diets per reference household over two weeks, in comparative terms (see Table 3), the most expensive diet on average was the HD (274.95 USD; 95% CI: 274.85–275.05), followed by the CD (261.84 USD; 95% CI: 261.62–262.06), with a very similar cost to FD1 (259.43 USD; 95% CI: 259.30–259.55), and then VD (256.96 USD; 95% CI: 256.90–257.03). The lowest costs were found for the CID (248.29 USD; 95% CI: 248.06–248.52) and FD2 (248.37 USD; 95% CI: 248.26–248.48). Significant differences were observed between the costs of the diets, except in some cases (between FD2 and CID).

**Table 3:** Cost and affordability of diets

Model diets	Current Diet (CD)	Current Isocaloric Diet (CID)	Healthy Diet (HD)	EAT Lancet Diets		
				Flexitarian Diet 1 (FD1)	Flexitarian Diet 2 (FD2)	Vegan Diet (VD)
Cost per household biweekly, in AR$	16872.98	16000.03	17717.73	16717.49	16005.09	16558.84
CI 95%	(16858.90–16887.05)	(15985.31–16014.75)	(17711.17–17724.29)	(16709.64–16725.33)	(15998.1–16012.08)	(16554.72 - 16562.96)
Cost per household biweekly, in USD	261.84	248.29	274.95	259.43	248.37	256.96
CI 95%	(261.62–262.06)	(248.06–248.52)	(274.85–275.05)	(259.30–259.55)	(248.26–248.48)	(256.90–257.03)
Cost per person (2000 kcal)* daily, in USD	3.77	3.83	4.26	4.03	3.88	4.01
CI 95%	(3.76–3.77)	(3.82–3.83)	(4.25–4.26)	(4.02–4.03)	(3.87–3-88)	(4.00–4.01)
Affordability, in %	55.9	53.0	58.7	55.4	53.0	54.8
CI 95%	(55.84–55.93)	(52.95–53.04)	(58.66–58.71)	(55.35–55.40)	(52.99–53.04)	(54.83–54.86)

*Cost per person daily standardized to an intake of 2000 kcal (assimilated to a 45-year-old woman), the results show lower cost for the current diet (differing from the other cost measures) because the biweekly household current diet includes a slightly higher calorie intake than the other model diets. CI, Confidence Interval; N.A., Not applicable; AR$, Argentine pesos; USD, United States Dollars. Source: Own estimation

The current diets appeared to have greater variability in their costs than diets based on the GAPA and EAT-Lancet recommendations. While it was observed that the maximum cost of CID and HD were very similar, approximately 75% of the CID diets had a lower cost than the minimum cost of HD. The lowest variability in cost corresponded to the VD, which could be related to the lower number of products in the diet (see Figure A.1 in Additional file 1).

In terms of the affordability of diets, defined as the percentage of monthly average household income needed to purchase a diet, Table 3 shows that 53.0% of the income was required to cover the cost of CID or FD1, 54.8% for VD, 55.4% for FD1, 55.9% for CD and 58.7% to afford the HD.

Regarding the contribution of the food groups to the cost of the diets, the results in Table 4 show some differences between model diets. In the HD and FD1, the main contribution came from fruits and vegetables which represented approximately one-third of the total cost of these diets (26.0% and 30.3% respectively). Dairy foods (21.0% and 19.1% respectively) and animal protein sources (20.6% and 15.8% respectively) also made a significant contribution, while tubers or starchy vegetables were the group that contributed the least (1.0% and 1.9% respectively).

**Table 4:** Food group contribution to cost

Model diets	Current Diet (CD)	Current Isocaloric Diet (CID)	Healthy Diet (HD)	EAT Lancet Diets		
				Flexitarian Diet 1 (FD1)	Flexitarian Diet 2 (FD2)	Vegan Diet (VD)
Food groups - contribution to cost, in %						
Fruits and vegetables	8.2	8.4	26.0	30.3	35.8	34.5
CI 95%	(8.2–8.2)	(8.4–8.4)	(26.0–26.0)	(30.3–30.4)	(35.8–35.8)	(34.4–34.5)
Tubers or starchy vegetables	1.4	1.3	1.0	1.9	2.0	1.8
CI 95%	(1.4–1.4)	(1.3–1.3)	(1.0–1.0)	(1.9–1.9)	(2.0–2.0)	(1.8–1.8)
Grains	6.7	6.7	7.9	9.5	10.2	25.5
CI 95%	(6.7–6.7)	(6.7–6.7)	(7.9–7.9)	(9.5–9.5)	(10.2–10.2)	(25.5–25.5)
Legumes	0.6	0.6	2.9	1.8	2.8	5.4
CI 95%	(0.6–0.6)	(0.6–0.6)	(2.9–2.9)	(1.8–1.8)	(2.8–2.8)	(5.4–5.4)
Dairy foods	16.7	17.0	21.0	19.1	13.7	0.0
CI 95%	(16.7–16.7)	(17.0–17.0)	(21.0–21.0)	(19.0–19.1)	(13.7–13.7)	N.A.
Animal protein sources	19.9	18.9	20.6	15.8	8.6	0.0
CI 95%	(19.8–19.9)	(18.9–18.9)	(20.6–20.7)	(15.8–15.9)	(8.6–8.7)	N.A.
Unsaturated oils - Nuts and seeds	2.7	2.4	5.0	13.2	18.1	22.8
CI 95%	(2.7–2.7)	(2.4–2.4)	(5.0–5.0)	(13.2–13.2)	(18.0–18.1)	(22.8–22.8)
Discretionary foods	33.2	33.4	8.0	3.1	3.0	1.3
CI 95%	(33.2–33.3)	(33.3–33.4)	(7.9–8.0)	(3.1–3.1)	(3.0–3.0)	(1.3–1.3)
Non-sugary beverages	9.2	9.7	7.5	5.3	5.9	6.5
CI 95%	(9.2–9.2)	(9.7–9.8)	(7.5–7.5)	(5.3–5.3)	(5.9–5.9)	(6.5–6.5)
Alcohol*	1.5	1.6	0.0	0.0	0.0	0.0
CI 95%	(1.5–1.5)	(1.6–1.6)	N.A.	N.A.	N.A.	N.A.
Supplement**	0.0	0.0	0.0	0.0	0.0	2.3
CI 95%	N.A.	N.A.	N.A.	N.A.	N.A.	(2.3–2.3)

*Alcohol products were only included in the current diet of adults

**Supplements were only included in the vegan diet. CI, Confidence Interval; N.A., Not applicable. Source: Own estimation

Similarly, the food group that contributed the most to the cost of the FD2 and VD were fruits and vegetables (35.8% and 34.5% respectively). In FD2 unsaturated oils - nuts and seeds also contributed significantly (18.1%) while tubers or starchy vegetables were the group that contributed the least (2.0%). Meanwhile, in VD, the grains group also had a significant contribution to the cost (25.5%) while discretionary foods contributed the least (1.3%).

In contrast, in the CD and CID, the food group that contributed the most to the cost was discretionary foods (33.2% and 33.4%), followed by animal protein sources (19.9% and 18.9%) and dairy foods (16.7% and 17.0%). On the other hand, legumes made the smallest contribution, with the same percentage in both (0.6%).

## Discussion

This study shows that the HD is the most expensive on average (and the least affordable) while the least expensive (and most affordable) are the CID and the FD2 (the version of the flexitarian diet with fewer animal protein sources included). Fruits and vegetables are the food group that contributes the most to the higher cost of HD. In terms of affordability, it was found that between 53% and 59% of the average income is needed to cover the cost of food. These findings are highly relevant for several reasons.

First, the higher cost of HD is a trend previously observed in Argentina, regardless of the methodology used to analyze it [13, 19]. Similar results were also found in some other countries [14, 16]^30^– [32], however, research based on the INFORMAS Protocol in Australia [33], Mexico [11], and Brazil [15] concluded that healthier diets could be accessed at a lower cost. It is important to note that the comparability of results between studies may be affected by different definitions of what constitutes a healthy diet and what constitutes a current diet for the cultural context in each country [11]. For example, the Australian research included takeaways and alcohol in the current diet, while for Argentina, takeaways were not included in the current diet. Likewise, variations in the composition of current diets are influenced by differing food habits [34].

The research showed that the food groups that contribute the most to total energy intake in HD are grains, unsaturated oils, nuts and seeds, and fruits and vegetables. The food group that contributes the most to the cost of HD is fruits and vegetables, which is consistent with a previous study conducted in Argentina [19]. This aspect is crucial, as food prices and affordability have been highlighted as one of the main reasons for following an unhealthy diet, and this could partly explain why less than half of the daily recommended amount of fruits and vegetables is consumed [18]. Subsidies or tax exemptions for fruits and vegetables and/or excise taxes on unhealthy or environmentally harmful products are recognised policies to promote healthier and more sustainable dietary patterns.

Second, it highlights that cost remains a significant barrier to accessing a healthier diet in Argentina, consistent with similar findings worldwide. A study of the Food and Agriculture Organization of the United Nations (FAO) [35] concluded that even the lowest-cost healthy diets are more expensive than the international poverty line, costing nearly five times as much as the most energy-efficient diets. As a result, they estimated that approximately 3 billion people worldwide lack the income necessary to afford the lowest-cost healthy diets recommended by national governments. In Argentina, 28.2% of the population considers their diet unhealthy, and price and accessibility dimensions were cited as the main reasons [18]. Moreover, the affordability measure is over 53% for all the diet models (and it almost reaches 59% for the HD) which means that the percentage of an average income that is needed to cover the cost of the diets is higher than the threshold (52%) frequently used as a reference for food expenses income share in low-income countries [36, 37].

Third, the average cost of FD2 is not significantly different from that of CID, suggesting that there are dietary options that can reduce greenhouse gas emissions and provide health benefits without increasing food expenses. This result is in line with those of the EAT-Lancet commission [7] and, at a country level, with studies from México [11], Australia [12] and a previous study carried out in Argentina [13, 22]. However, the latter finding raises questions about the feasibility of promoting a shift in consumption patterns in a country like Argentina which is characterized by high red and processed meat consumption [38, 39]. The evidence shows that in countries with historically high meat consumption, the recent trends in consumption have followed different directions. For example, the average per capita meat consumption decreased by 17.4 g per day between 2008 and 09 and 2018-19 in the United Kingdom [40]. Meanwhile, in the Low Countries, the status quo persists when it comes to maintaining a meat-rich diet [41]. Cultural acceptability still seems to be a major challenge for the transition towards more environmentally sustainable diets in Argentina.

Given that a multiplicity of economic, social, and environmental factors influencing meat demand have been identified, many of which are difficult to address through direct political interventions, it has been suggested that consumer awareness could help mitigate high meat consumption by influencing food preferences [42]. For example, the Eatwell Guides from the United Kingdom highlight plant-based sources in the protein section and recommend to eat less red and processed meats [43], and the Mediterranean diet pyramid has also incorporated aspects of sustainability and biodiversity, promoting local, seasonal, fresh, and minimally processed foods, as well as an increase in plant-based proteins and a responsible and conscious consumption of white meats and fish [44]. With these references in mind and considering that the HD was more expensive than the other diets, updating the Argentine dietary guidelines with a perspective of environmental and economic sustainability stands out as a key first step. It is also relevant that these dietary guidelines address cultural differences that may exist in the country.

Another challenge of promoting EAT-Lancet diets is their ability to meet the requirements for certain essential micronutrients, particularly vitamin B12, iron, calcium, and zinc [45]. Flexitarian options could serve as suitable alternatives; however, it would still be necessary to ensure that these diets cover all essential nutrients, especially for certain population groups where these are critical, such as children and pregnant women. In some cases, supplementation might be necessary, such as vitamin B12 in vegan versions, due to its absence in the foods included in these diets [3].

As a limitation of this study, it is worth mentioning that the research does not refer to the actual consumption of the population. Instead, it is based on a modelling methodology that considers the most recent data available about consumption patterns in Argentina, National Dietary Guidelines recommendations, and EAT-Lancet recommendations. In addition, using EAT-Lancet recommendations as parameters for designing more sustainable diets without measuring the environmental impact of the modeled diets could be mentioned as a shortfall; this will be included in a future publication by the research team. This research also does not specifically analyse the health effects of different model diets, something that would be very relevant to explore in future research. Moreover, although the price data used may seem somewhat outdated, it was the most recent source available that included price information for all products in the dietary patterns at the time of the research. Another limitation related to the price data is that average prices for Greater Buenos Aires were used rather than those for the entire country. Given that approximately 40% of Argentina’s population lives in this region, we consider it to be a good proxy. In a recently published study [46], the differences in cost and affordability of current and healthy diets were specifically analysed by region of the country.

In spite of these limitations, this research proposes improvements in diet design over a previous version of the same team [19], as it uses the most recent information on consumption patterns of the population from a nutrition survey, rather than from household expenditure surveys, and introduces the analysis of model diets based on EAT-Lancet recommendation as a more environmentally friendly option. In addition, the diets were designed specifically for each of the four family members, taking into account their characteristics and the amounts to be consumed in a family context during a 14-day time period. Regarding the composition of the diets, the design included a larger number of food products to have more variability and feasible combinations that meet the parameters.

## Conclusions

In a context where reducing the environmental impact of food systems is essential, promoting diets with less red meat is crucial. In Argentina, a country with a high tradition of red meat consumption, this is culturally complex. This study showed that flexitarian diets with less animal protein sources and based on EAT-Lancet recommendations, proposed as more environmentally friendly options, can be less costly compared to healthy diets, and can be reached at a similar average cost to some current diets in Argentina. In this sense, it is necessary to update the Dietary Guidelines for the Argentine Population by incorporating a sustainability approach. It is also crucial to implement policies that reduce the cost of recommended foods, such as fruits and vegetables, to make these diets more affordable to the entire population.

A comprehensive and coordinated policy package is fundamental to creating healthier and more sustainable food environments. This package should include regulations on marketing targeted at children, the promotion of healthy school environments, clear front-of-package labeling, and the implementation of excise taxes on unhealthy and environmentally harmful products to increase their relative prices. These widely recognized strategies are effective in guiding consumers toward healthier and more sustainable dietary choices. Such policies are key to improving consumption patterns, closing persistent food and nutrition gaps, and ensuring the accessibility, availability, and affordability of nutritious foods by addressing the physical, economic, and social barriers that perpetuate inequalities in access to adequate nutrition.

## Supplementary Information

Supplementary Material 1.

## Data Availability

Data is provided within the manuscript or supplementary information files. Also, the datasets used and/or analysed during the current study are available from the corresponding author upon request.

## Abbreviations

NCD: Non-communicable diseases
IPCC: Intergovernmental Panel on Climate Change
UPF: Ultra-processed foods
INFORMAS: International Network for Food and Obesity/Non-communicable Diseases Research, Monitoring and Action Support
CD: Current diet
HD: Healthy diet
FD: Flexitarian diet
VD: Vegan diet
CID: Current isocaloric diet
GAPA: Spanish acronym for National Dietary Guidelines of Argentina
ENNyS2: Spanish acronym for Second National Nutrition and Health Survey
CHO: Carbohydrates
BMI: Body mass index
SARA: Spanish acronym for Food Registration and Analysis System
AR$: Argentine pesos
USD: United States Dollars
FAO: Food and Agriculture Organization of the United Nations
