# Supplementary material for: Cost and affordability implications of transitioning from current diets to National dietary guidelines and EAT-Lancet recommendations in Argentina: a modelling study
Source: Nutr J. 2025 Oct 22;24:161. doi: 10.1186/s12937-025-01212-7 (PMC12542274; doi:10.1186/s12937-025-01212-7)
Supplement: Supplementary file 1 — Supplementary Material 1. [file 12937_2025_1212_MOESM1_ESM.docx]

***Supplementary information***

Table A.1. Food products list, by INFORMAS and EAT-Lancet food groups.

| **Food product** | **Food group - INFORMAS** | **Food groups - EAT-LANCET** |
| --- | --- | --- |
| Banana | 1. Fruit | 1. Fruits and vegetables |
| Orange |  |  |
| Apple |  |  |
| Peach |  |  |
| Mandarin |  |  |
| Pear |  |  |
| Onion | 2. Vegetables |  |
| Tomato |  |  |
| Carrot |  |  |
| Tomato purée |  |  |
| Red pepper |  |  |
| Lettuce |  |  |
| Pumpkin |  |  |
| Garlic |  |  |
| Green or yellow pepper |  |  |
| Courgette |  |  |
| Chard |  |  |
| Green onion |  |  |
| Potato | 3. Starchy vegetables | 2. Tubers or starchy vegetables |
| Corn |  |  |
| Sweet potato |  |  |
| French bread | 4. Grains | 3. Grains |
| White rice |  |  |
| Dried noodles/pasta |  |  |
| Wheat flour |  |  |
| Breadcrumbs |  |  |
| "Empanada*" or pie pastry |  |  |
| Pre-pizza dough |  |  |
| Hot dog or hamburger bread, packaged |  |  |
| White bread, sliced |  |  |
| Bran bread | 5. Wholemeal Grains |  |
| Rolled oats |  |  |
| Whole wheat flour |  |  |
| Brown rice |  |  |
| Quinoa |  |  |
| Canned peas | 6.Legumes | 4. Legumes |
| Lentils |  |  |
| Beans |  |  |
| Soybeans |  |  |
| Fluid whole milk, fortified with vitamins A and D | 7. Milk | 5. Dairy foods |
| Fluid part-skimmed milk, fortified with vitamins A and D |  |  |
| Powdered whole milk powder, fortified with vitamins A and D |  |  |
| Flavored whole yoghurt (drinkable) | 8. Yoghurt |  |
| Non-fat yoghurt |  |  |
| Cheese (in feta) | 9. Cheese |  |
| Packaged grated cheese |  |  |
| Mozzarella Cheese |  |  |
| Cream Cheese |  |  |
| Whole cream cheese, spreadable |  |  |
| Non-fat cheese (port salut type) |  |  |
| Fat-free cream cheese spread |  |  |
| Chicken egg | 10. Meat and eggs | 6. Beef, lamb, and pork - Chicken and other poultry - Fish – Egg |
| Chicken without skin |  |  |
| Chicken with skin |  |  |
| Breaded chicken schnitzel (*milanesa**) |  |  |
| Chicken breast without skin |  |  |
| Minced beef (regular) |  |  |
| Breaded beef schnitzel (milanesa*) |  |  |
| Minced beef (low fat) |  |  |
| Roast (with bone) |  |  |
| Round steak |  |  |
| Shoulder |  |  |
| Canned tuna, natural |  |  |
| Hake |  |  |
| Sunflower oil | 11. Vegetable oils, nuts and seeds | 7. Unsaturated oils - Nuts and seeds |
| Olive oil |  |  |
| Edible oil mix |  |  |
| Walnut |  |  |
| Almond |  |  |
| Sunflower seed |  |  |
| Flax seed |  |  |
| Chia seed |  |  |
| Sesame seed |  |  |
| Salt | 12. Salt | 8. Discretionary foods |
| White sugar | 13. Sugar |  |
| Mayonnaise | 14. Fatty foods |  |
| Milk cream |  |  |
| Butter |  |  |
| Beef fat |  |  |
| Cooked ham | 15. Processed meats |  |
| Vienna sausage |  |  |
| “Chorizo*” |  |  |
| Beef burger |  |  |
| Salami |  |  |
| Mortadella |  |  |
| “Paleta*” (cold meat) |  |  |
| Plain sweet biscuits | 16. Cookies and pastries |  |
| Stuffed sweet biscuits |  |  |
| Bran or wholemeal biscuits |  |  |
| Water biscuits |  |  |
| Sweet biscuits with quince (*pepas**) |  |  |
| Stuffed pastries |  |  |
| Plain pastries |  |  |
|  |  |  |
| Argentine savory biscuits |  |  |
| Fruit jam | 17. Sweets |  |
| Light fruit jam |  |  |
| “Dulce de leche” |  |  |
| Cocoa powder with sugar |  |  |
| Cream ice cream |  |  |
| Chocolate “*alfajor**” |  |  |
| Chewy candies |  |  |
| Bouillon cubes | 18. Bouillon cubes and powders |  |
| Vinegar | 19. Sauces and dressings |  |
| Tomato-based sauce ready-to-eat |  |  |
| Mustard |  |  |
| French fries | 20. Snacks and appetizers |  |
| Green olives |  |  |
| Liquid sweetener with saccharine and cyclamate | 21. Artificial sweeteners |  |
| Juice powder with sugar | 22. Sugary beverages |  |
| Soda with sugar, cola flavor |  |  |
| Fruit juice |  |  |
| Instant coffee (powder or granulated for brewing) |  |  |
| "Yerba mate*" | 23. Non-sugary beverages | 9. Non-sugary beverages |
| Coffee prepared from ground coffee beans (filter coffee type) |  |  |
| Sparkling water |  |  |
| Light powdered juice |  |  |
| Black tea in tea bags |  |  |
| Bottled water |  |  |
| "Matecocido*" in tea bags |  |  |
| Water from public water supply or tap water, filtered (water filter or water dispenser) |  |  |
| Beer with alcohol | 24. Alcohol beverages | 10. Alcohol |
| Red wine |  |  |
| Vitamin B12 | 25. Supplement | 11. Supplement |

Note: *Spanish names.

Figure A.1. Cost distribution of the model diets


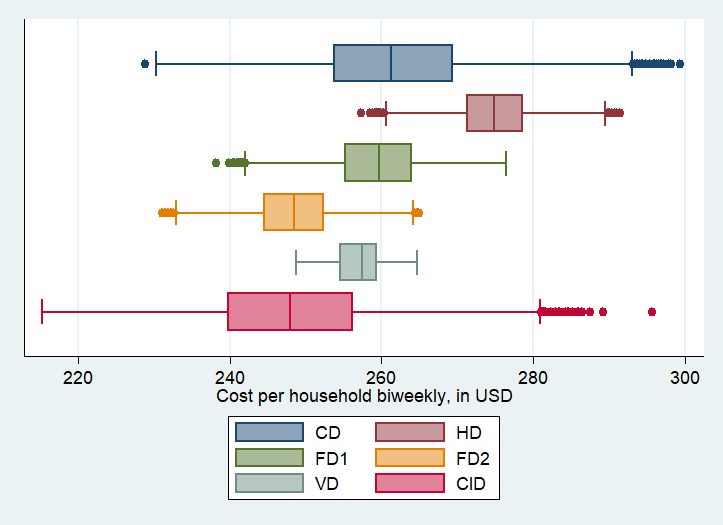
Note: USD, United States Dollars; CD, current diet; HD, healthy diet; FD1, flexitarian diet 1; FD2, flexitarian diet 2; VD, vegan diet; CID, current isocaloric diet. Source: Own estimation.
